# Supplementary material for: Phosphorylation regulates the binding of autophagy receptors to FIP200 Claw domain for selective autophagy initiation
Source: Nat Commun. 2021 Mar 10;12:1570. doi: 10.1038/s41467-021-21874-1 (PMC7946963; doi:10.1038/s41467-021-21874-1)
Supplement: Supplementary file 1 — Supplementary Information [file 41467_2021_21874_MOESM1_ESM.pdf]

Phosphorylation regulates the binding of autophagy receptors to FIP200 Claw domain for selective autophagy initiation

Zhou et al.

Supplementary Figures

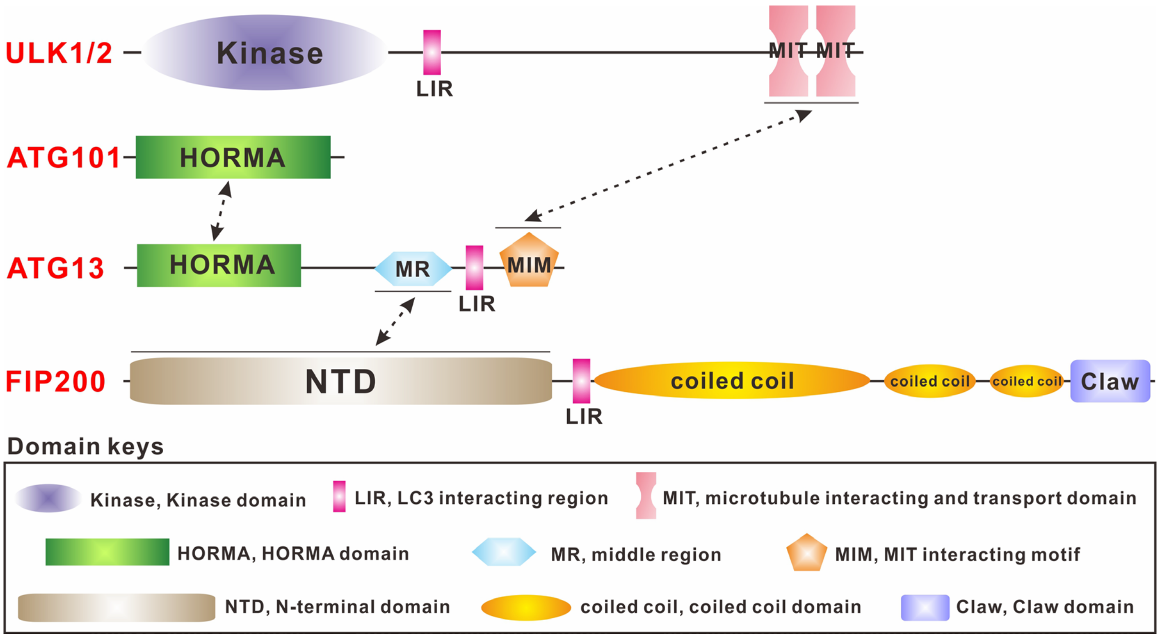

Supplementary Figure 1. The components and the related protein-protein interaction network of ULK complex. A schematic diagram showing the domain organizations of four ULK complex subunits, ULK1/2, ATG101, ATG13, and FIP200. In this drawing, the ATG101/ATG13, ATG13/ULK1/2 and ATG13/FIP200 interactions are further highlighted and indicated by two-way arrows.

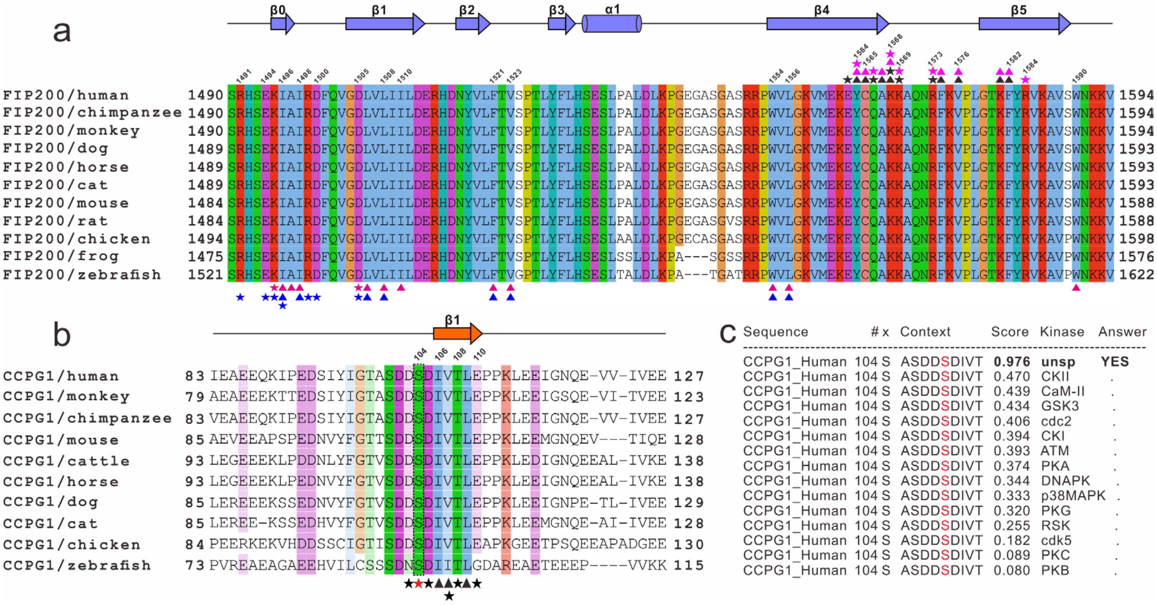

Supplementary Figure 2. Sequence alignment analyses of the FIP200 Claw region and the FIR2 region of CCPG1. (**a**) Structure-based sequence alignment of FIP200 Claw domain from different species. The conserved residues are highlighted by colors using software Jalview2.10.5 (http://www.jalview.org/). In this alignment, the conserved residues that are involved in hydrophobic interactions with p-CCPG1 FIR2 are highlighted with black triangles, and the polar interactions are highlighted with black stars. Meanwhile, the conserved residues that are involved in hydrophobic interactions with p-Optineurin LIR are highlighted with pink triangles, and the polar interactions are highlighted with pink stars on the top of the sequence alignment. For the dimerization of apo-form FIP200 Claw domain, the interface residues that are crucial for polar interactions are labeled with magenta stars, and hydrophobic interactions are labeled with magenta triangles on the bottom of the sequence alignment. In contrast, for the dimerization of FIP200 Claw domain in the FIP200 Claw/p-CCPG1 complex, the interface residues that are crucial for polar interactions are labeled with blue stars, and hydrophobic interactions are labeled with blue triangles. (**b**) Structure-based sequence

alignment of CCPG1 FIR2 regions from different species. The conserved residues that are involved in hydrophobic interactions with FIP200 Claw are highlighted with black triangles, and the polar interactions are highlighted with black stars, and the potentially phosphorylated S104 site is highlighted with a red star. (**c**) The phosphorylation site predication of CCPG1 FIR2 region by the NetPhos 3.1 server (http://www.cbs.dtu.dk/services/NetPhos/). The prediction score (a value above 0.500 indicate positive predictions) suggests that the S104 residue in CCPG1 FIR2 is potential phosphorylation site, which is colored in red. The string "unsp" stands for non-specific kinase prediction, and the string "YES" indicates positive predictions.

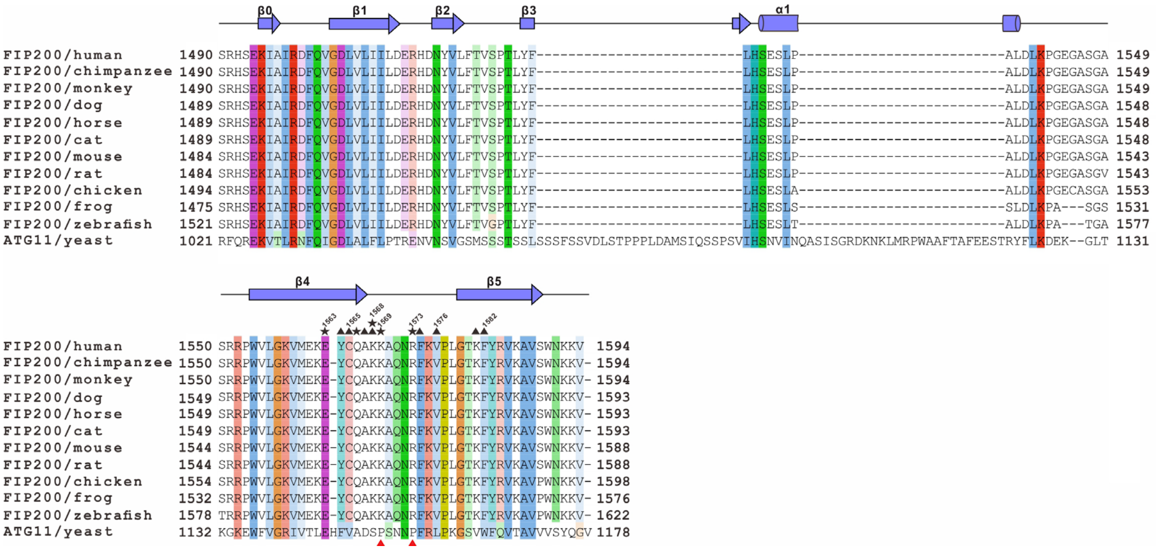

Supplementary Figure 3. Structure-based sequence alignment of FIP200 Claw regions from different species and the corresponding C-terminal region of yeast ATG11. The conserved residues are highlighted by colors using software Jalview2.10.5 (http://www.jalview.org/). In this alignment, the conserved residues that are involved in hydrophobic interactions and polar interactions with p-CCPG1 FIR2 are highlighted with black triangles and black stars, respectively. In addition, the two Pro residues

corresponding to the positively charged K1569 and R1573 residues of human FIP200, which are essential for interacting with p-CCPG1 FIR2, are further marked and highlighted with red triangles. The missing of these two critical Lys and Arg residues in ATG11 indicates that the C-terminal region of ATG11 is different from FIP200 Claw in binding to autophagy receptors.

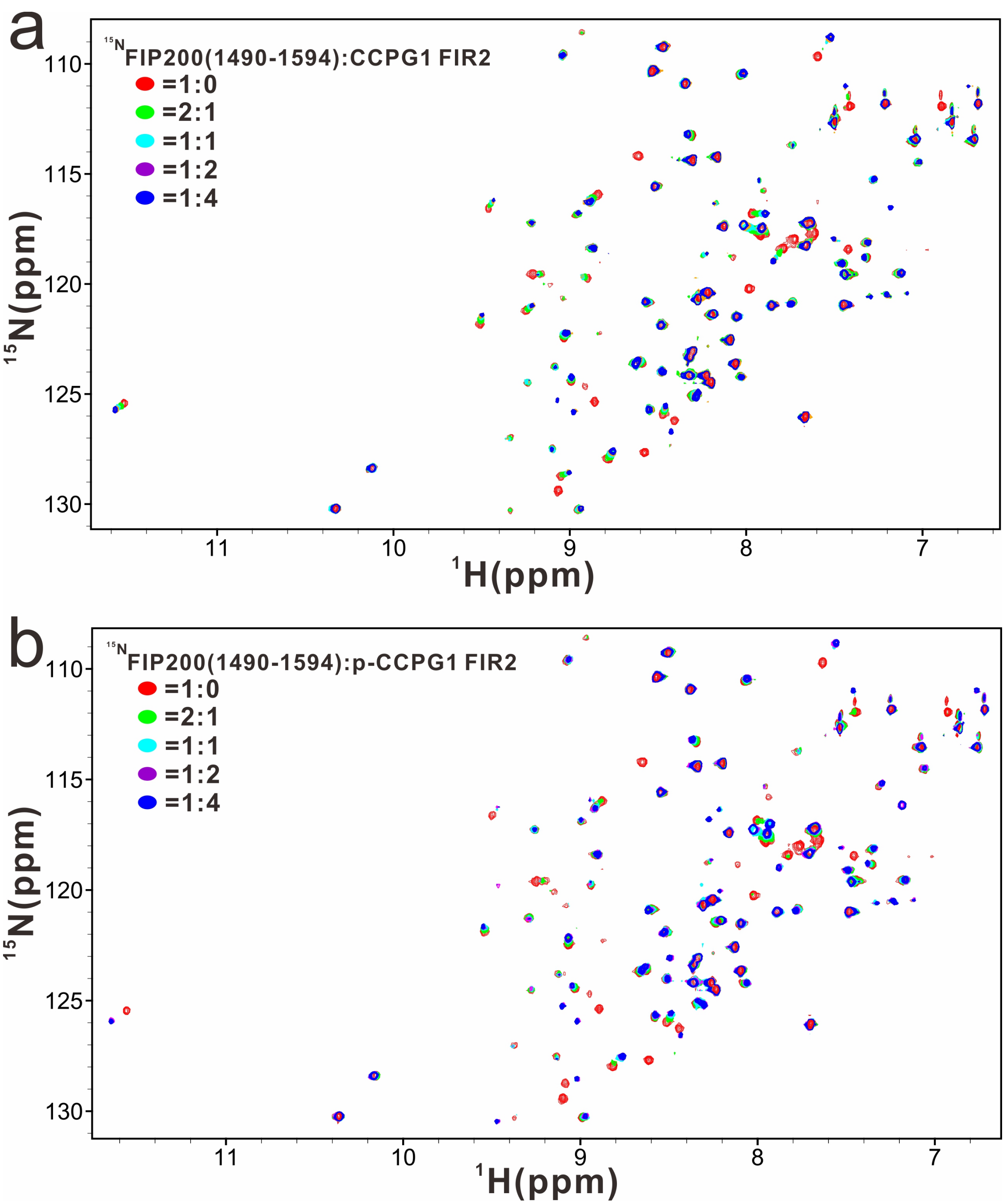

Supplementary Figure 4. NMR-based characterizations of the interactions of FIP200 Claw with CCPG1 FIR2 and p-CCPG1 FIR2. (**a**) Superposition plots of the ^{1}H-^{15}N HSQC spectra of FIP200 Claw domain titrated with the un-labeled CCPG1 FIR2 peptides at different molar ratios. (**b**) Superposition plots of the ^{1}H-^{15}N HSQC spectra of

FIP200 Claw domain titrated with the un-labeled p-CCPG1 FIR2 peptides at different molar ratios.

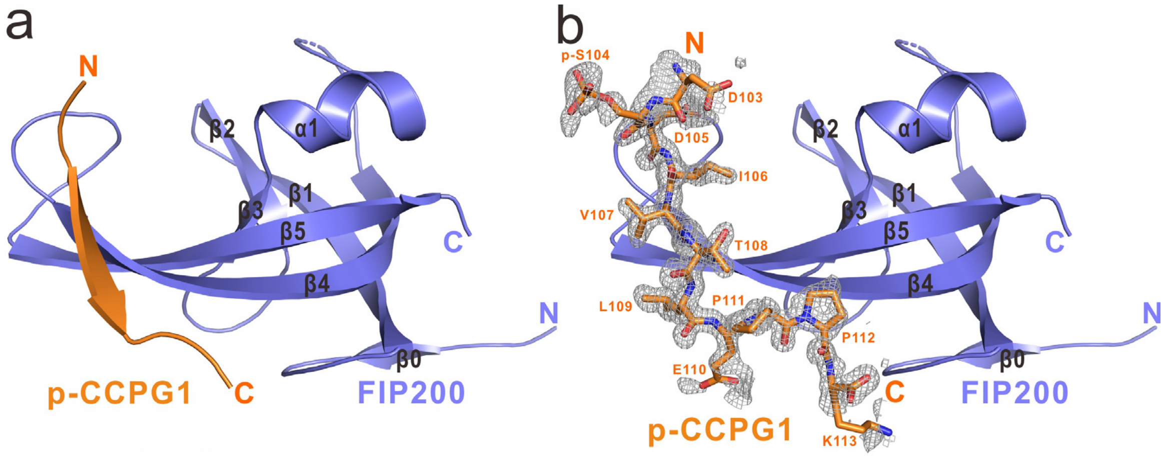

Supplementary Figure 5. Structure analyses of the monomeric FIP200 Claw/p-CCPG1 complex in an asymmetric unit. (**a**) Ribbon diagram showing the overall structure of the monomeric FIP200 Claw/p-CCPG1 FIR2 complex with a 1:1 binding stoichiometry in an asymmetric unit. (**b**) The F_O-F_C map of the p-CCPG1 FIR2 showing that the densities of 11 FIR2 residues ('DpSDIVTLEPPK') can be clearly assigned. The map is calculated by omitting a p-CCPG1 FIR2 peptide from the final PDB file, and is contoured at 2.0σ.

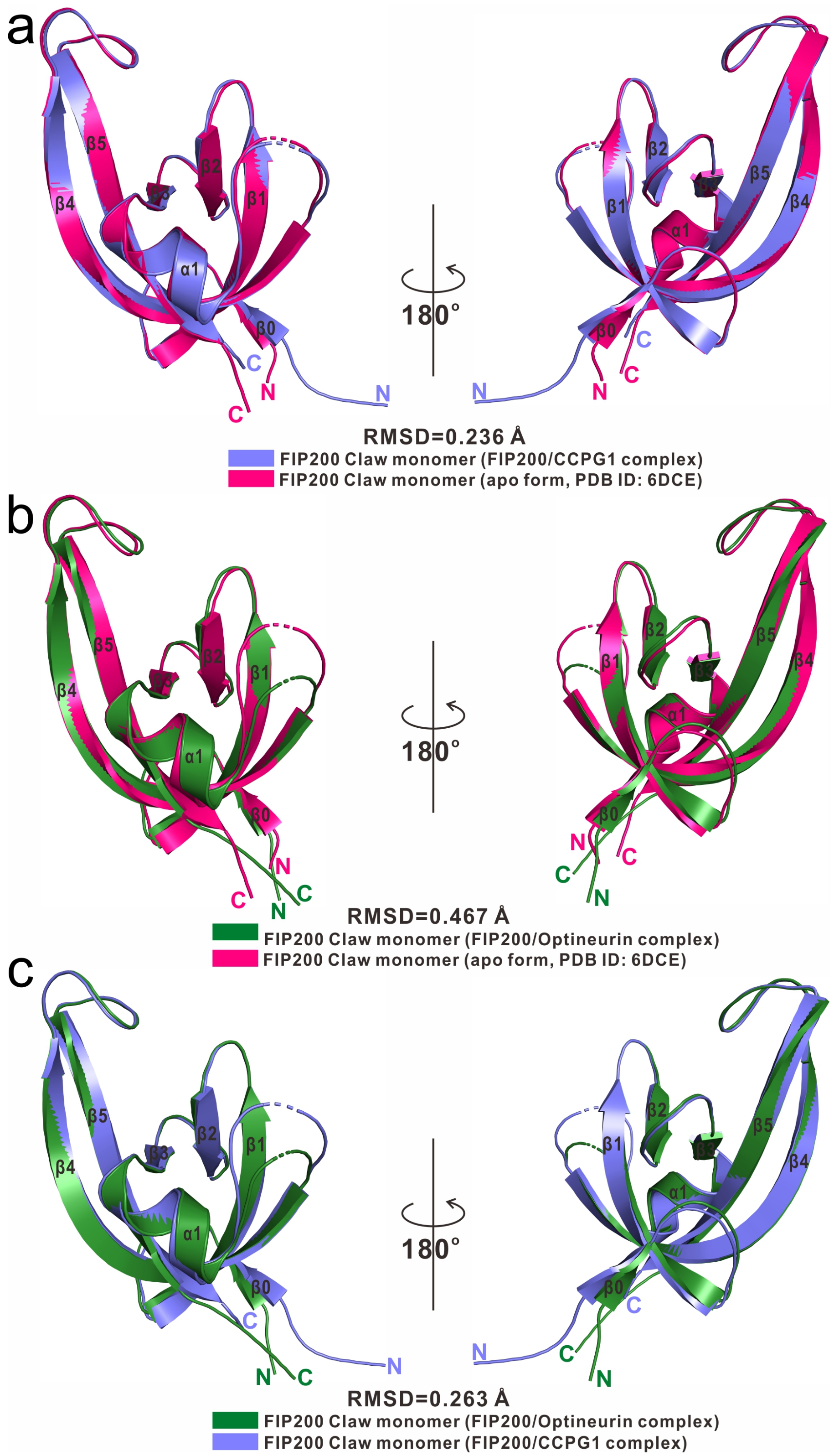

Supplementary Figure 6. The comparisons of the overall structures of the monomeric FIP200 Claw domain in the apo form and in the FIP200 Claw/p-CCPG1

FIR2 and FIP200 Claw/p-Optineurin LIR complexes. (**a** and **b**) Ribbon representation showing the structural comparison of apo-form FIP200 Claw monomer (hot pink, PDB ID: 6DCE [http://www.rcsb.org/structure/6DCE]) with the representative FIP200 Claw monomer in the FIP200 Claw/p-CCPG1 FIR2 complex (slate) (**a**), or in the FIP200 Claw/p-Optineurin LIR complex (green) (**b**). (**c**) Ribbon representation showing the structural comparison of the FIP200 Claw monomers in the FIP200 Claw/p-CCPG1 FIR2 complex (slate) and the FIP200 Claw/p-Optineurin LIR complex (green).

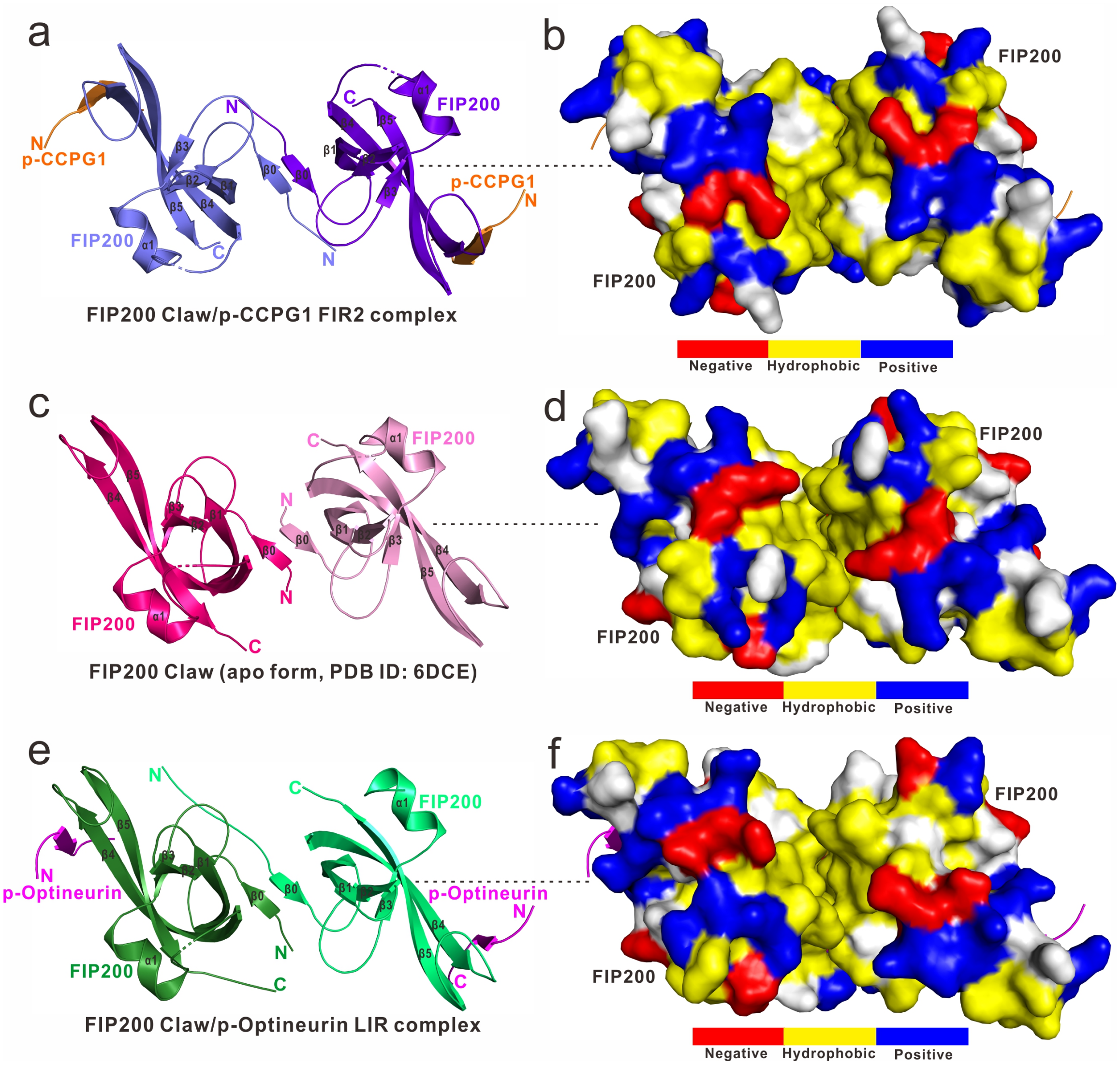

Supplementary Figure 7. Structural analyses of the overall conformations of the dimeric FIP200 Claw domain in the apo form and in the FIP200 Claw/p-CCPG1 FIR2 and FIP200 Claw/p-Optineurin LIR complexes. (**a**, **c** and **e**) Ribbon diagram showing the overall structures as well as the steric arrangements of dimeric FIP200 Claw domain in the FIP200 Claw/p-CCPG1 FIR2 complex (**a**), in the apo form (PDB ID: 6DCE [http://www.rcsb.org/structure/6DCE]) (**c**)**,** or in the FIP200 Claw/p-Optineurin LIR complex (**e**). (**b**, **d** and **f**) The combined surface representation and the ribbon-stick model showing the hydrophobic binding interface between two monomeric FIP200 Claw domains in the FIP200 Claw/p-CCPG1 FIR2 complex (**b**), in the apo form (**d**), or in the

FIP200 Claw/p-Optineurin LIR complex (**f**). The Claw domain is showed in surface representation colored by amino acid types. Particularly, in the surface model of FIP200 Claw, the hydrophobic amino acid residues are drawn in yellow, the positively charged residues in blue, the negatively charged residues in red, and the uncharged polar residues in grey. Apparently, the binding of p-CCPG1 FIR2 to the dimeric FIP200 Claw domain is likely to open up a hydrophobic pocket between the two FIP200 Claw monomers.

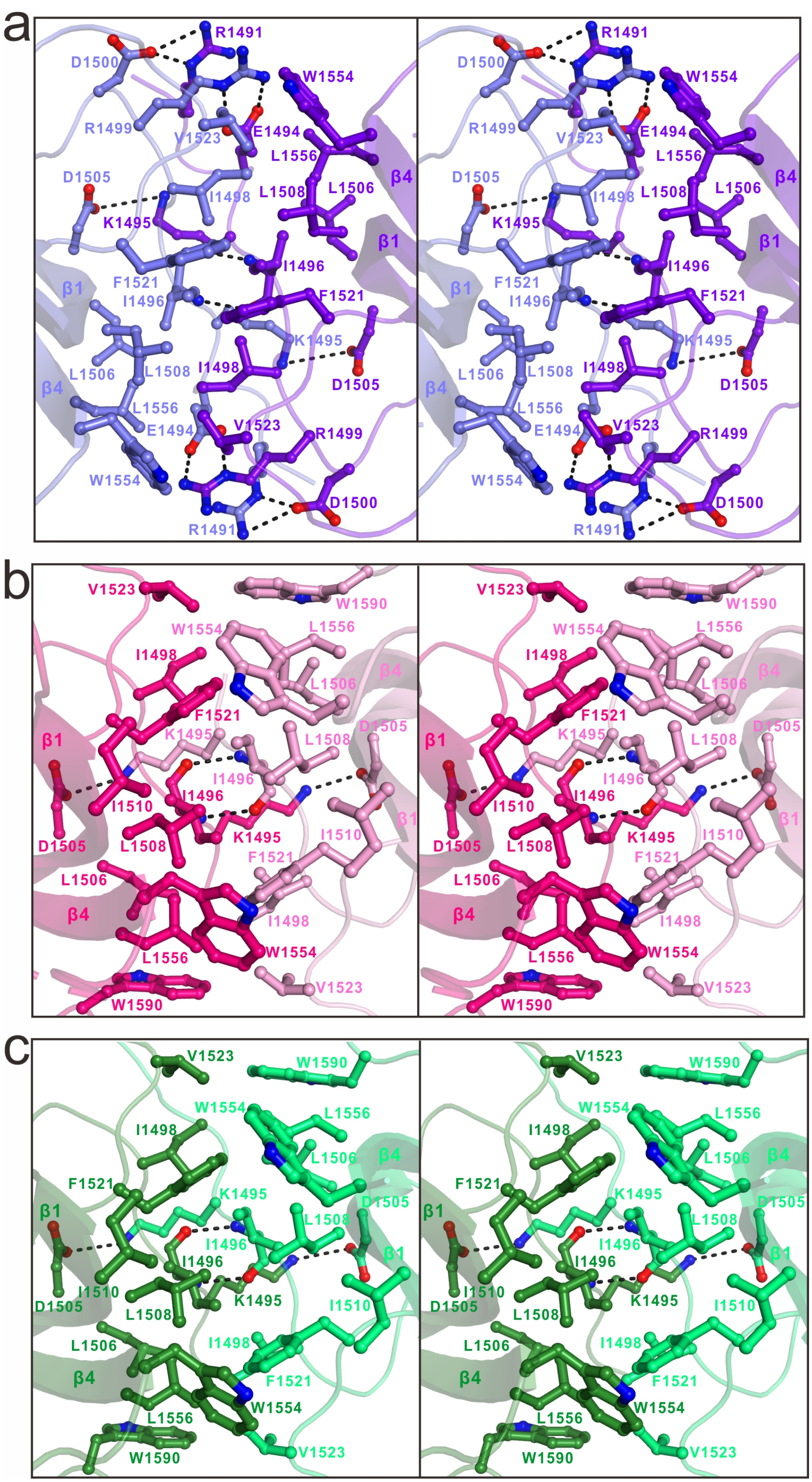
a
R1491
D1500
W1554
R1499
V1523
E1494
L1556
β4
D1505
L1508
L1506
I1498
K1495
β1
I1496
F1521
K1495
I1498
D1505
L1506
L1508
L1556
E1494
V1523
R1499
W1554
D1500
R1491
b
V1523
W1590
W1554
L1556
I1498
β4
L1506
F1521
D1505
K1495
L1508
I1496
I1510
D1505
L1508
K1495
I1510
F1521
L1506
I1498
W1554
L1556
V1523
W1590
c
V1523
W1590
W1554
L1556
I1498
L1506
F1521
K1495
D1505
L1508
I1496
β1
I1510
D1505
L1508
K1495
I1498
F1521
L1506
β4
W1554
L1556
V1523
W1590

Supplementary Figure 8. Detailed structural analyses of the dimerization interfaces of FIP200 Claw dimer in the apo form and in the FIP200 Claw/p-CCPG1 FIR2 and FIP200 Claw/p-Optineurin LIR complexes. (**a-c**) Stereo view of the ribbon-stick model showing the detailed interactions in the dimerization interface of FIP200 Claw domain in the FIP200 Claw/p-CCPG1 FIR2 (**a**), in the apo form (PDB ID: 6DCE [http://www.rcsb.org/structure/6DCE]) (**b**), or in the FIP200 Claw/p-Optineurin LIR (**c**). The relevant hydrogen bonds and salt bridges involved in the binding are shown as dotted lines.

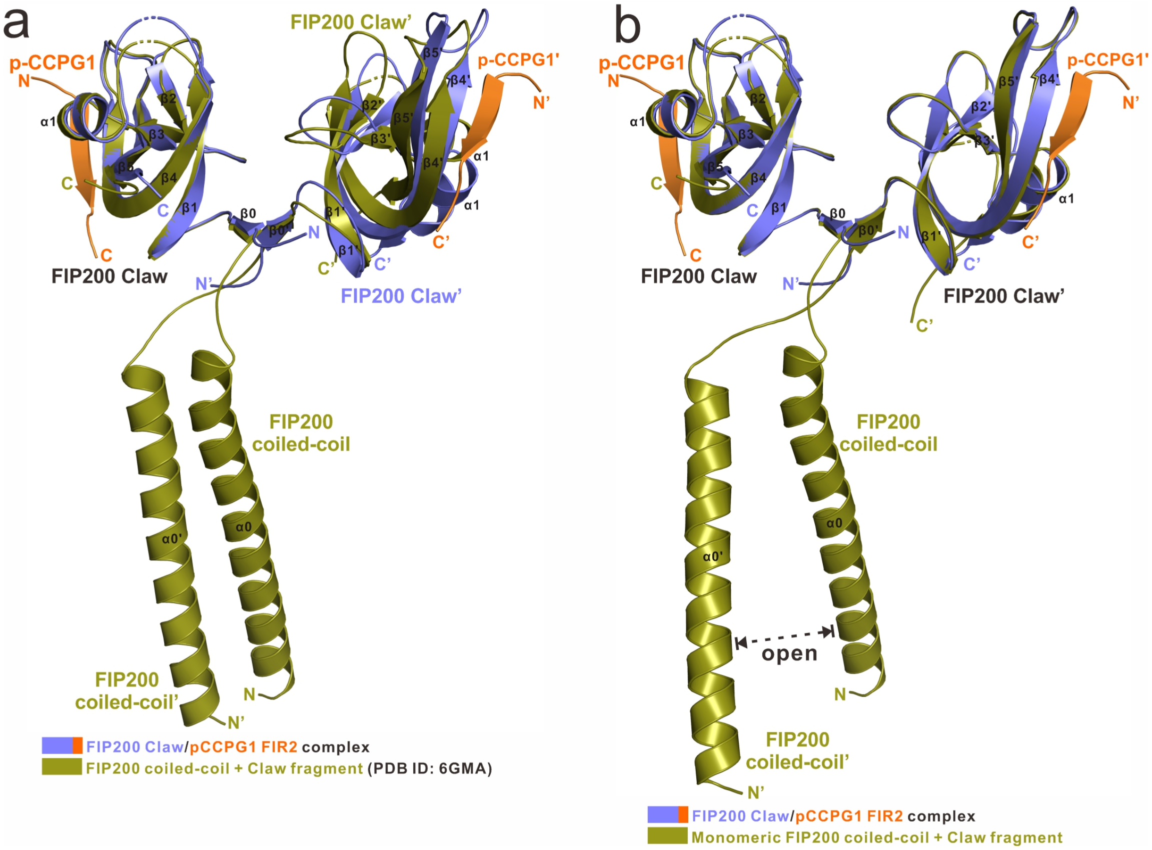

Supplementary Figure 9. The comparisons of the FIP200 Claw/p-CCPG1 FIR2 complex structure with the determined structure of a FIP200 C-terminal fragment including the Claw domain and the preceding coiled-coil domain. (**a** and **b**) Ribbon

representations showing the structural comparisons of the FIP200 Claw/p-CCPG1 FIR2 complex (slate/orange) with the apo-form FIP200 fragment containing the Claw domain and the preceding coiled-coil domain (olive, PDB ID: 6GMA [http://www.rcsb.org/structure/6GMA]). In panel **a**, the two dimeric structures are overlaid by aligning selected one Claw monomer in these two structures. In panel **b**, the two monomeric FIP200 C-terminal fragments are individually aligned to the two Claw domains of the FIP200 Claw/p-CCPG1 FIR2 complex.

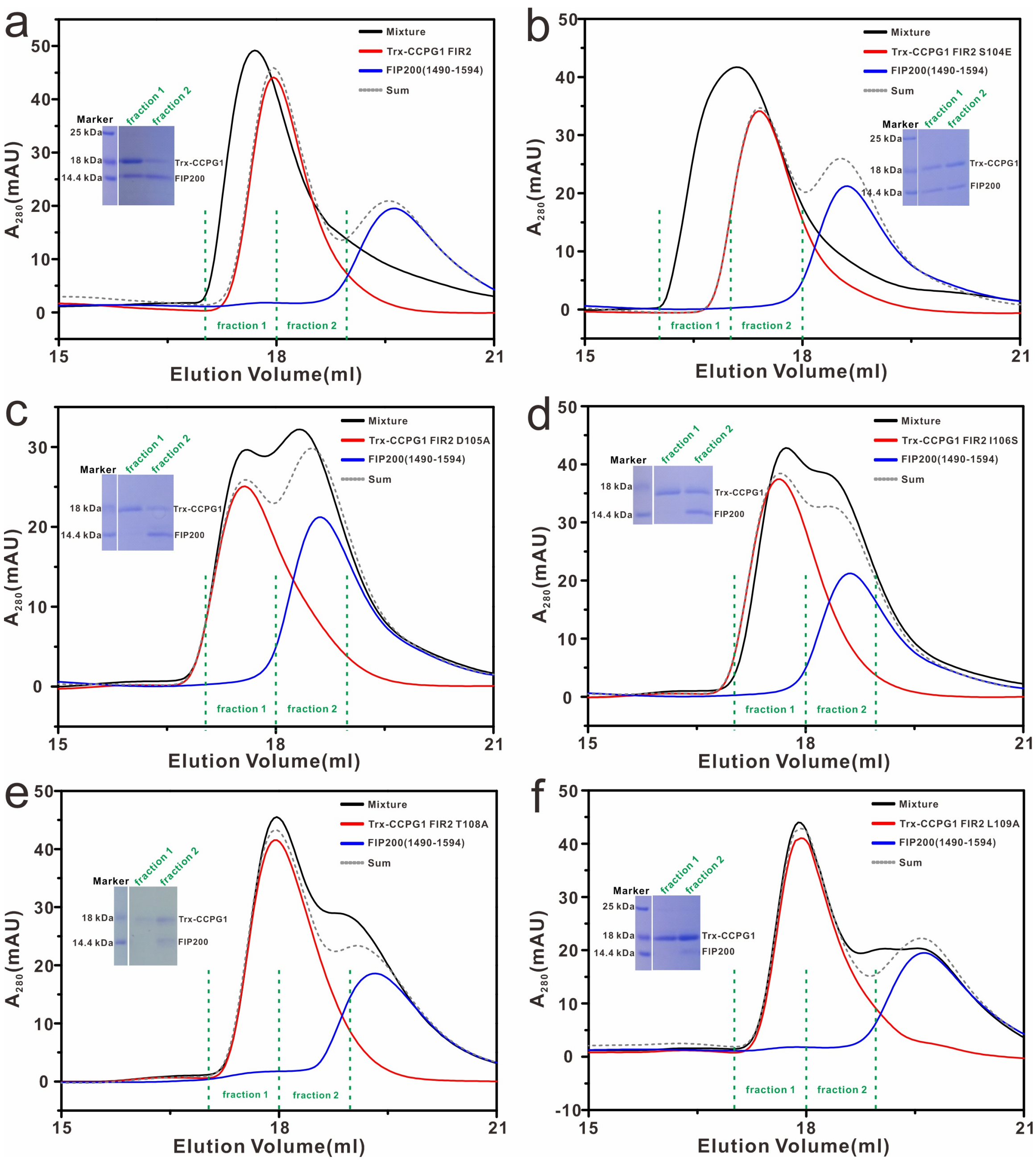

Supplementary Figure 10. Validations of the FIP200 Claw/p-CCPG1 FIR2 complex structure by mutations of key interface residues in CCPG1. (**a-f**) Analytical gel filtration chromatography coupled with SDS-PAGE analyses of the interactions of FIP200 Claw domain with CCPG1 FIR2 (**a**), the phosphomimetic S104E mutant (**b**), D105A mutant (**c**), I106S mutant (**d**), T108A mutant (**e**), and L109A mutant (**f**). In each panel, the insert shows the SDS-PAGE combined with Coomassie-blue staining analyses

of the protein components of the indicated "fraction 1" and "fraction 2" fractions collected from the analytical gel filtration chromatography experiment of the FIP200 Claw/CCPG1 mixture (the black curve). These SDS-PAGE experiments were repeated twice independently with similar results. Source data are provided as a Source Data file.

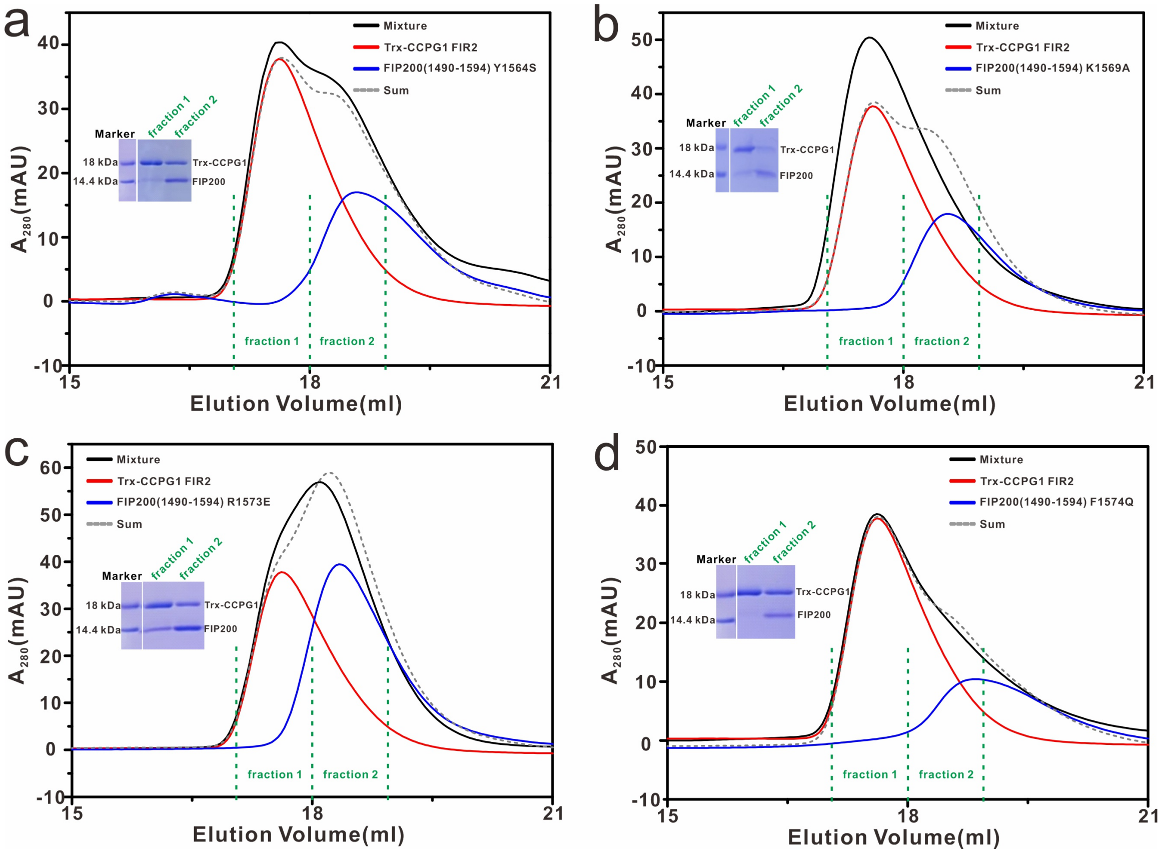

Supplementary Figure 11. Validations of the FIP200 Claw/p-CCPG1 FIR2 complex structure by mutations of key interface residues in FIP200 Claw domain. (**a-d**) Analytical gel filtration chromatography coupled with SDS-PAGE analyses of the interactions of CCPG1 FIR2 with FIP200 Claw Y1564A mutant (**a**), K1569A mutant (**b**), R1573E mutant (**c**), and F1574Q mutant (**d**). These results further validate the key interface residues observed in the FIP200 Claw/p-CCPG1 FIR2 complex structure. In each panel, the insert shows the SDS-PAGE combined with Coomassie-blue staining

analyses of the protein components of the indicated “fraction 1” and “fraction 2” fractions collected from the analytical gel filtration chromatography experiment of the CCPG1 FIR2/FIP200 Claw variant mixture (the black curve). These SDS-PAGE experiments were repeated twice independently with similar results. Source data are provided as a Source Data file.

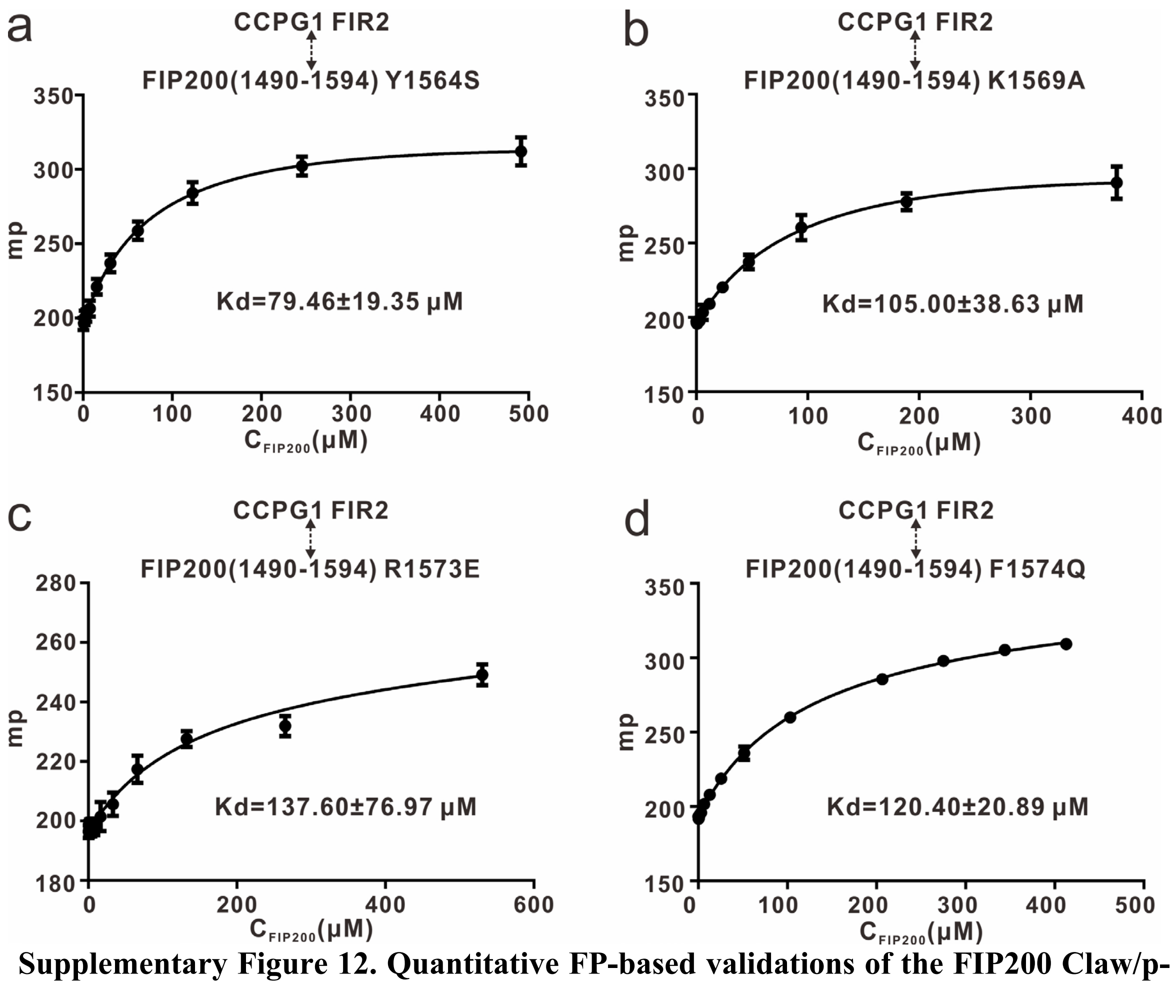

Supplementary Figure 12. Quantitative FP-based validations of the FIP200 Claw/p-CCPG1 FIR2 complex structure by mutations of key interface residues in FIP200 Claw. (**a-d**) FP-based measurements of the binding affinities of CCPG1 FIR2 with the FIP200 Claw Y1564S mutant (**a**), K1569A mutant (**b**), R1573E mutant (**c**), and F1574Q mutant (**d**). *K*d values are the fitted dissociation constants with standard errors, when

using the one-site binding model to fit the FP data. These FP experiments were performed in triplicate and error bars represented the standard deviation here (n=3). Source data are provided as a Source Data file.

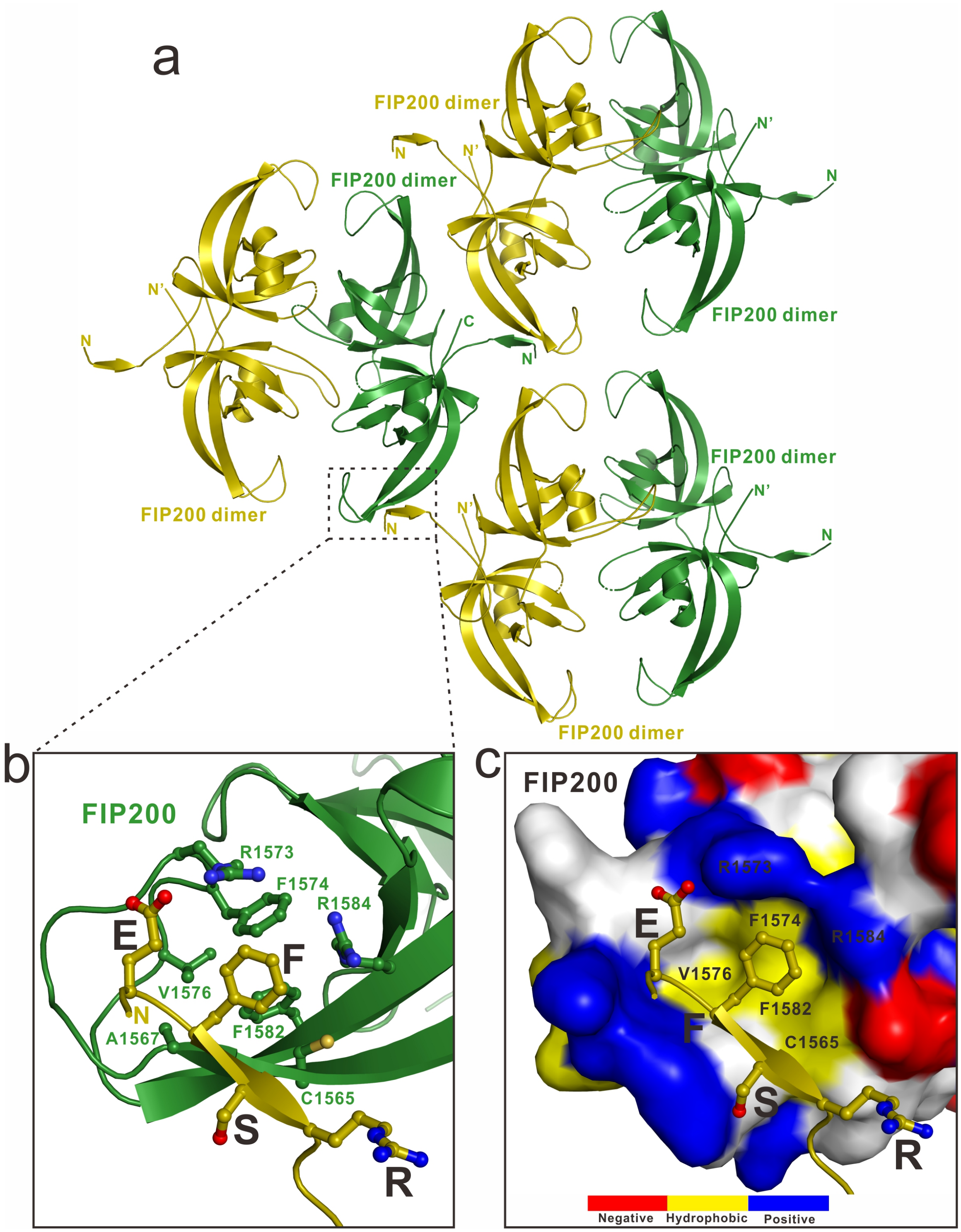

Supplementary Figure 13. Structural analyses of the apo-form structure of FIP200 Claw domain determined in this study. (**a**) Ribbon diagram showing the overall structure as well as the crystal packing of FIP200 Claw dimer in the determined apo-form

crystal structure of FIP200 Claw domain. Notably, a short β-strand formed by the remaining sequences of the N-terminal cleaved 3C protease site of a FIP200 Claw monomer directly packs with a nearby Claw molecule from another FIP200 Claw dimer for the crystal packing. (**b**) The ribbon-stick model showing the enlarged view of a selected region where the Glu-Phe residues from the remaining sequences of the N-terminal cleaved 3C protease site in one FIP200 Claw binds to the "LHP" region in a neighboring FIP200 Claw monomer. (**c**) The combined surface representation and the ribbon-stick model showing the detailed binding surface between FIP200 Claw and the Glu-Phe residues from the remaining sequences of the N-terminal cleaved 3C protease site as displayed in panel **b**. In this drawing, the FIP200 Claw domain is showed in surface representation colored by amino acid types. Specifically, the hydrophobic amino acid residues in the surface model of FIP200 Claw are drawn in yellow, the positively charged residues in blue, the negatively charged residues in red, and the uncharged polar residues in gray.

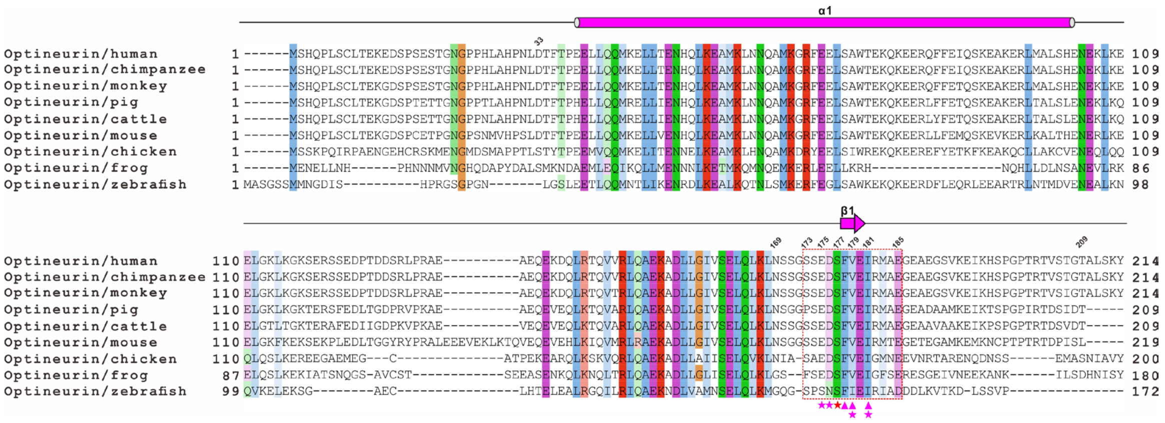

Supplementary Figure 14. Structure-based sequence alignment of Optineurin(1-214) regions from different species. The conserved residues are highlighted by colors using software Jalview2.10.5 (http://www.jalview.org/). In this alignment, the conserved

residues of Optineurin that are involved in the hydrophobic interactions with FIP200 Claw are highlighted with magenta triangles, the polar interactions are highlighted with magenta stars, and the phosphorylated S177 site by TBK1 is highlighted with a red star.

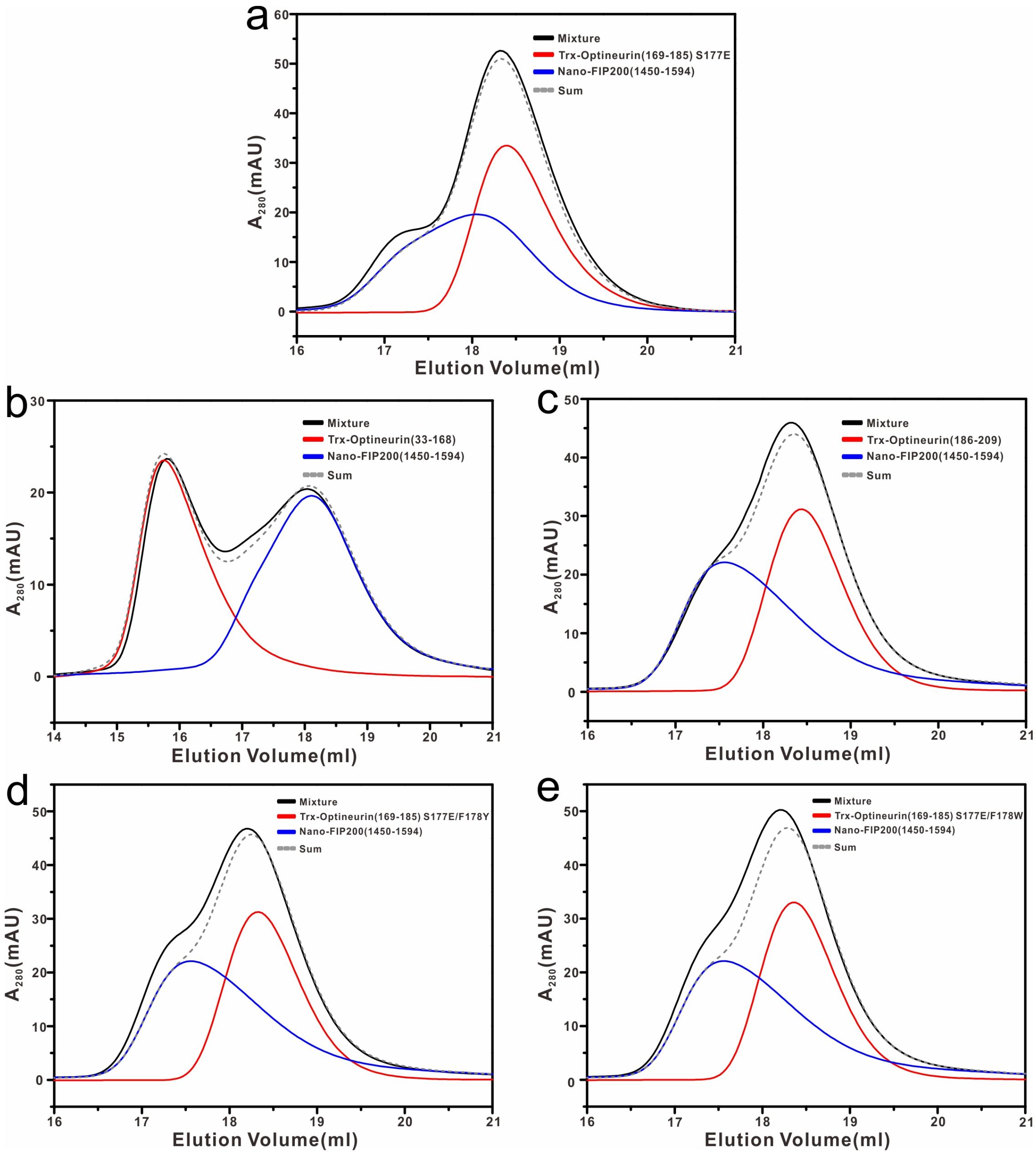

Supplementary Figure 15. Biochemical characterizations of the interactions between FIP200(1450-1594) and different Optineurin variants. (a-e) Analytical gel filtration

chromatography analyses of the interactions of FIP200(1450-1594) with the phosphomimetic Optineurin(169-185) S177E mutant (**a**), the Optineurin(33-168) fragment (**b**), the Optineurin(186-209) fragment (**c**), the Optineurin(169-185) S177E/F178Y mutant (**d**), and the Optineurin (169-185) S177E/F178W mutant (**e**). Source data are provided as a Source Data file.

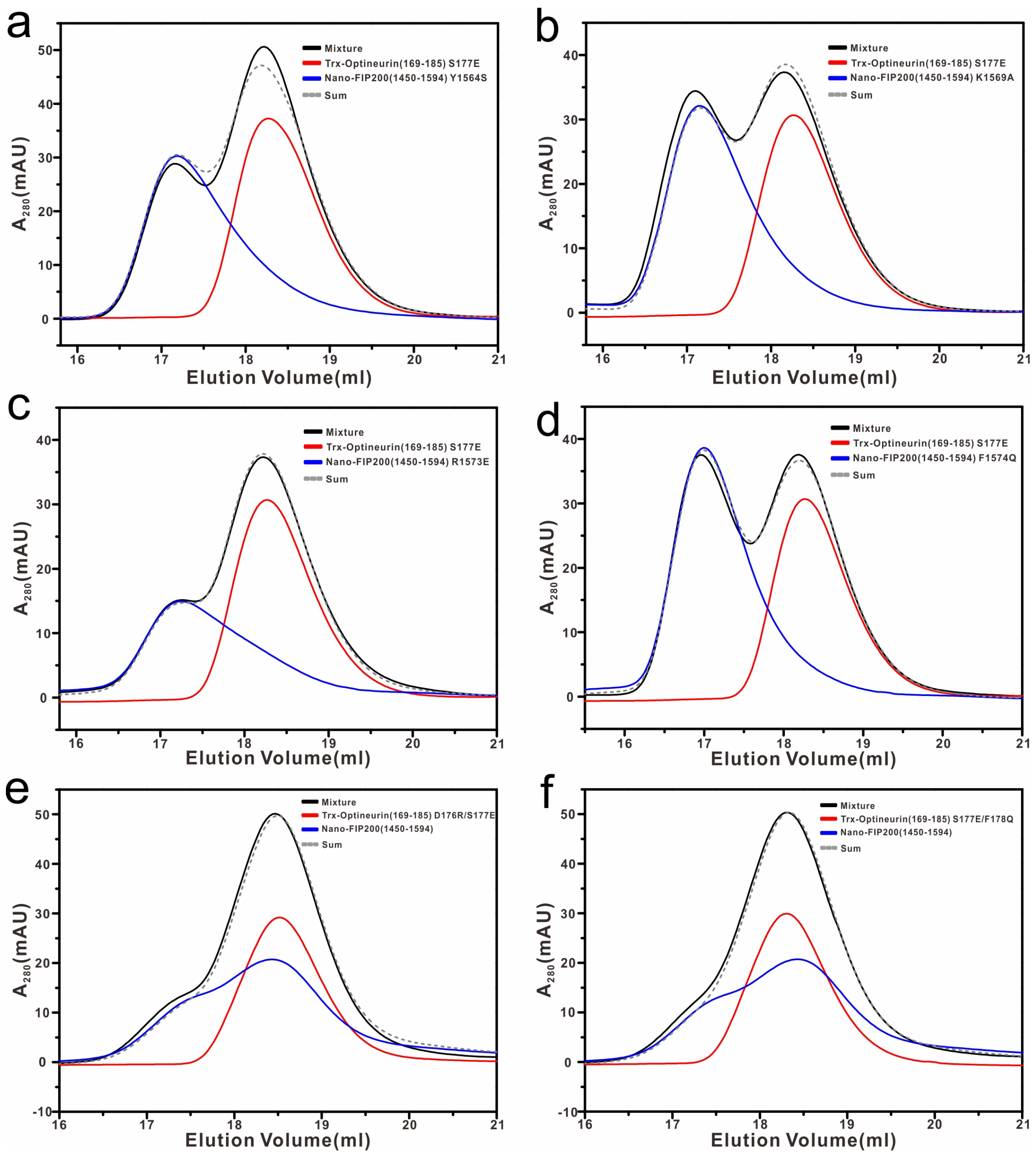

Supplementary Figure 16. Validations of the FIP200 Claw/p-Optineurin LIR complex structure by mutations of key binding interface residues. (**a-d**) Analytical gel filtration chromatography analyses of the interactions of the phosphomimetic Optineurin(169-185) S177E mutant with the FIP200(1450-1594) Y1564S mutant (**a**), K1569A mutant (**b**), R1573E mutant (**c**), and F1574Q mutant (**d**). (**e** and **f**) Analytical gel filtration chromatography analyses of the interactions of FIP200(1450-1594) with the Optineurin(169-185) D176R/S177E mutant (**e**), and S177E/F178Q mutant (**f**). Source data are provided as a Source Data file.

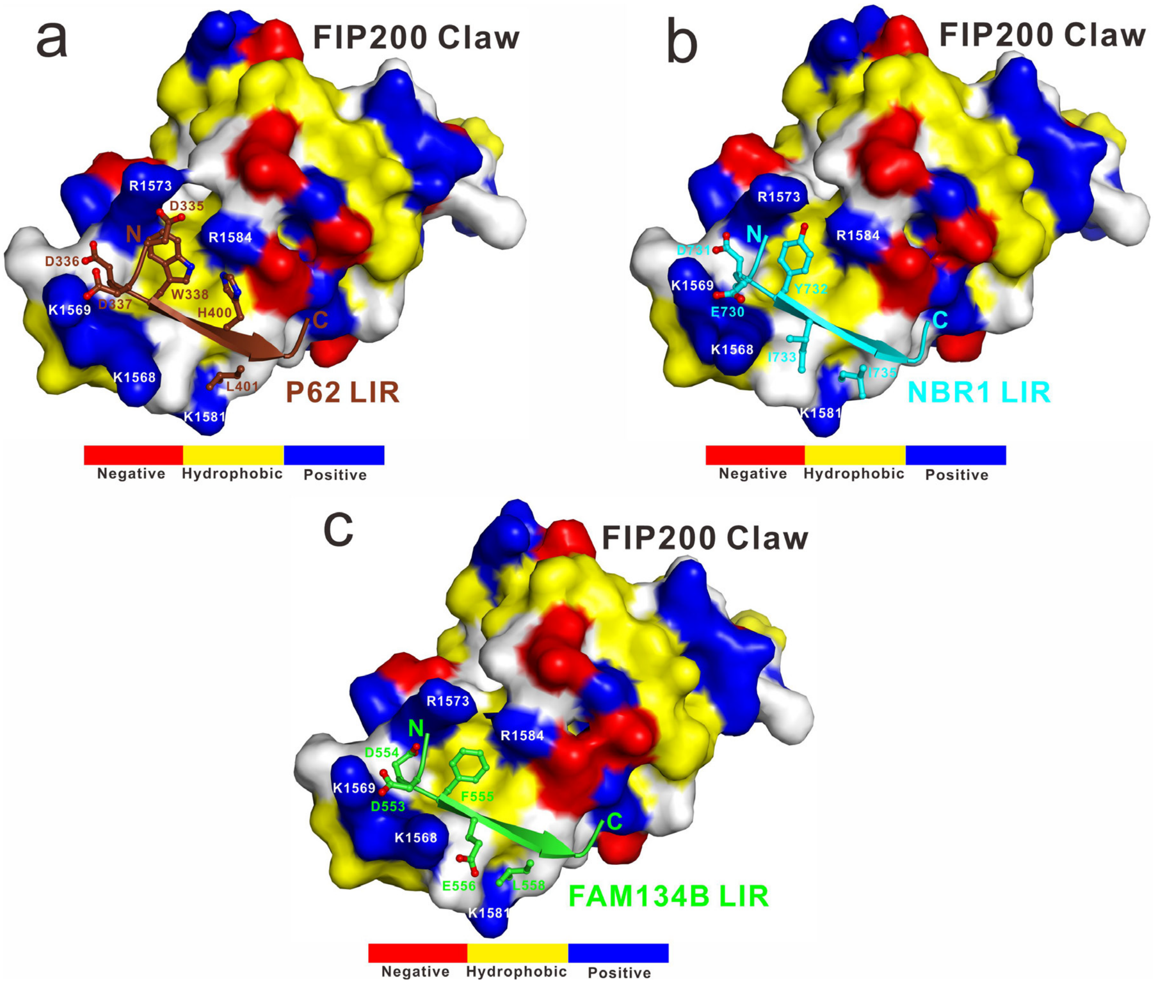

Supplementary Figure 17. Structural modeling analyses of the potential interactions between FIP200 Claw and the LIR regions of P62, NBR1 and FAM134b. (a-c) The combined surface representation and the ribbon-stick model showing the detailed binding surface between FIP200 Claw and P62 LIR (**a**), NBR1 LIR (**b**), or FAM134B LIR (**c**) in a structural model of the FIP200 Claw/P62 LIR complex, the FIP200 Claw/NBR1 LIR complex, or the FIP200 Claw/FAM134B LIR complex. In these drawings, the LIR regions of P62, NBR1 and FAM134b are displayed in the ribbon-stick model, and the FIP200 Claw domains are showed in the surface representation colored by amino acid types. Specifically, the hydrophobic amino acid residues in the surface model of FIP200 Claw are drawn in yellow, the positively charged residues in blue, the negatively charged

residues in red, and the uncharged polar residues in gray. These structural models were initially generated based on the determined crystal structure of the FIP200 Claw/p-CCPG1 FIR2 complex or the FIP200 Claw/p-Optineurin LIR complex using PyMOL (http://www.pymol.org/), and were further refined using the YASARA energy minimization server (http://www.yasara.org/minimizationserver.htm) to increase the model accuracy

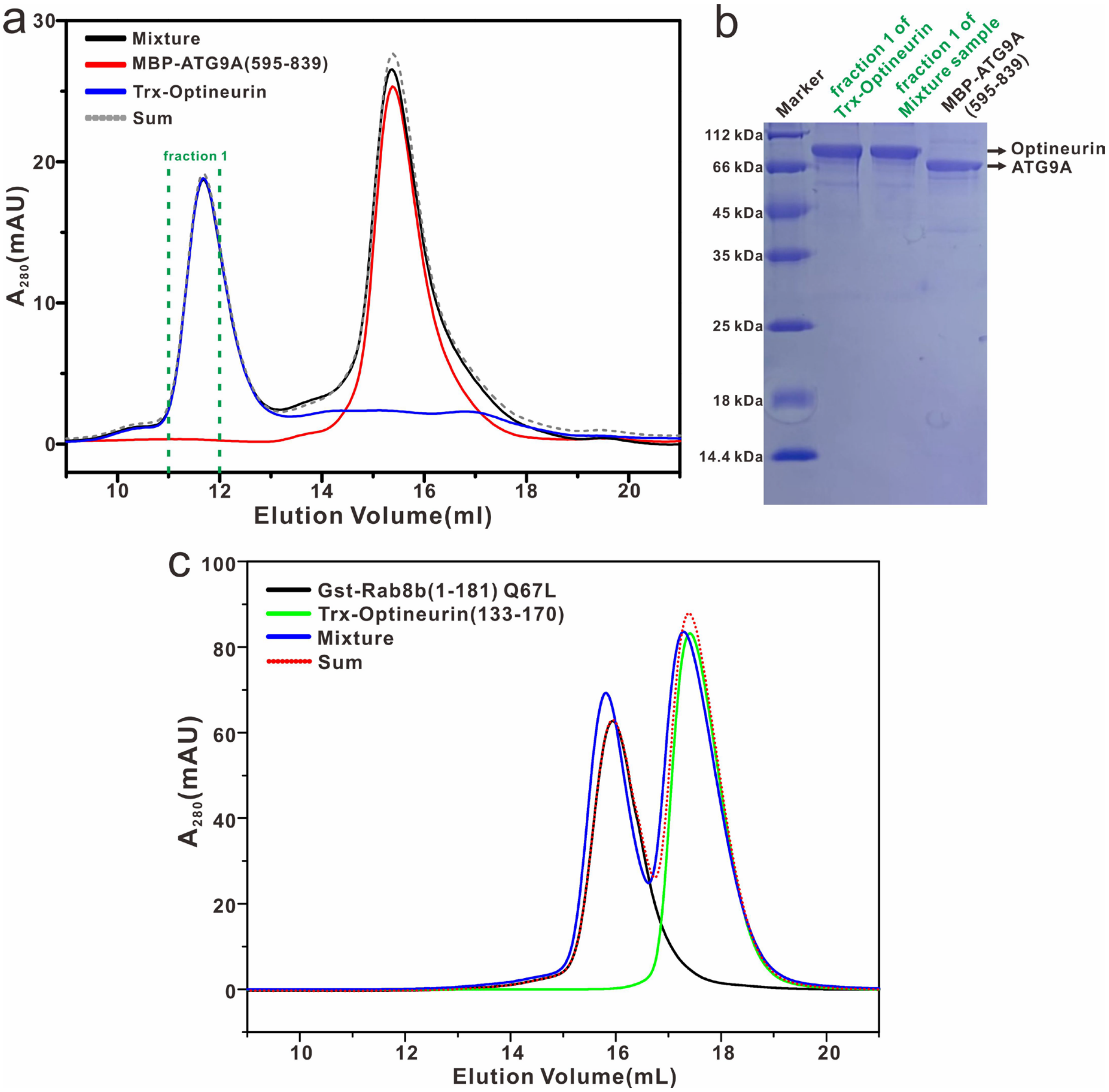

Supplementary Figure 18. Biochemical characterizations of the interactions of Optineurin with ATG9A and Rab8b. (**a**) Analytic gel filtration chromatography-based analyses of the interaction of MBP-tagged ATG9A(595-839) with full-length Optineurin. (**b**) The SDS-PAGE combined with Coomassie-blue staining analysis of the protein components of the input MBP-tagged ATG9A(595-839) protein as well as the indicated "fraction 1" fractions collected from the analytic gel filtration chromatography experiments of Trx-tagged Optineurin (the blue curve) and the MBP-ATG91A(595-839)/Trx-Optineurin mixture (the black curve). This experiment was repeated twice

independently with similar results. (**c**) Analytic gel filtration chromatography-based analysis of the interaction between the constitutively active Rab8b Q67L mutant and the Optineurin(133-170) fragment. Source data are provided as a Source Data file

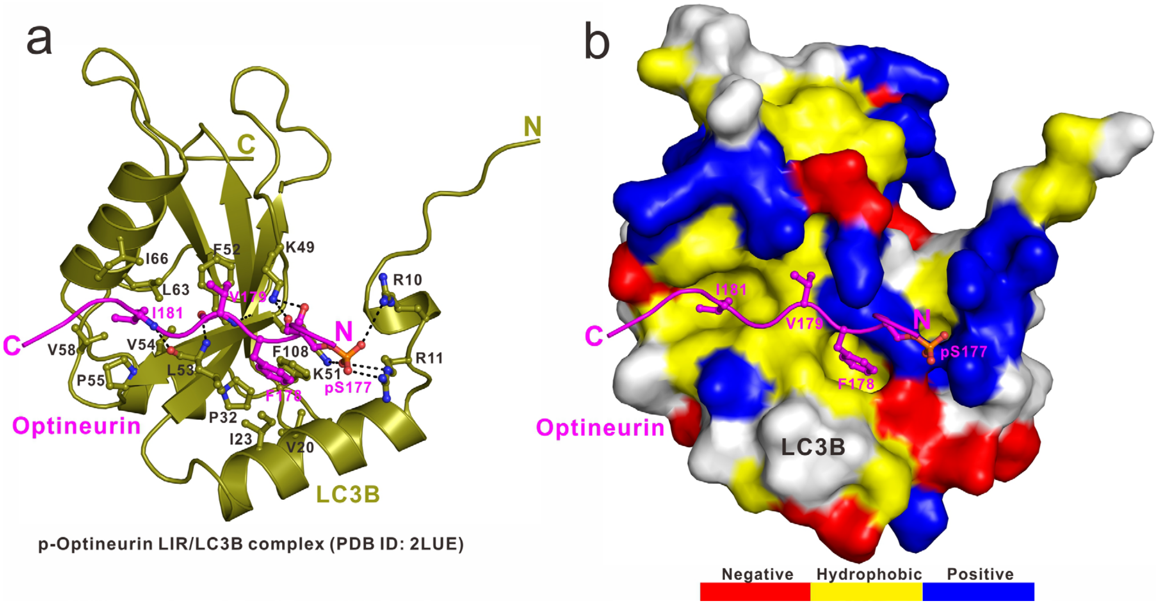

Supplementary Figure 19. Structural analyses of the LC3B/p-Optineurin LIR complex. (**a**) The ribbon-stick representation showing the detailed interactions between LC3B and p-Optineurin LIR in the previously determined LC3B/p-Optineurin LIR complex structure (PDB ID: 2LUE [http://www.rcsb.org/structure/2LUE]). In this drawing, the side chains as well as relevant backbone groups of the key interface residues are shown in the stick-ball mode, and the hydrogen bonds involved in the binding are shown as dotted lines. (**b**) The combined surface representation and the ribbon-stick model showing the molecular interface between LC3B and p-Optineurin LIR. In this drawing, the p-Optineurin LIR is displayed in the ribbon-stick model, while the LC3B is showed in surface representation colored by amino acid types. Specifically, the hydrophobic amino acid residues in the surface model of LC3B are drawn in yellow, the

positively charged residues in blue, the negatively charged residues in red, and the uncharged polar residues in gray.

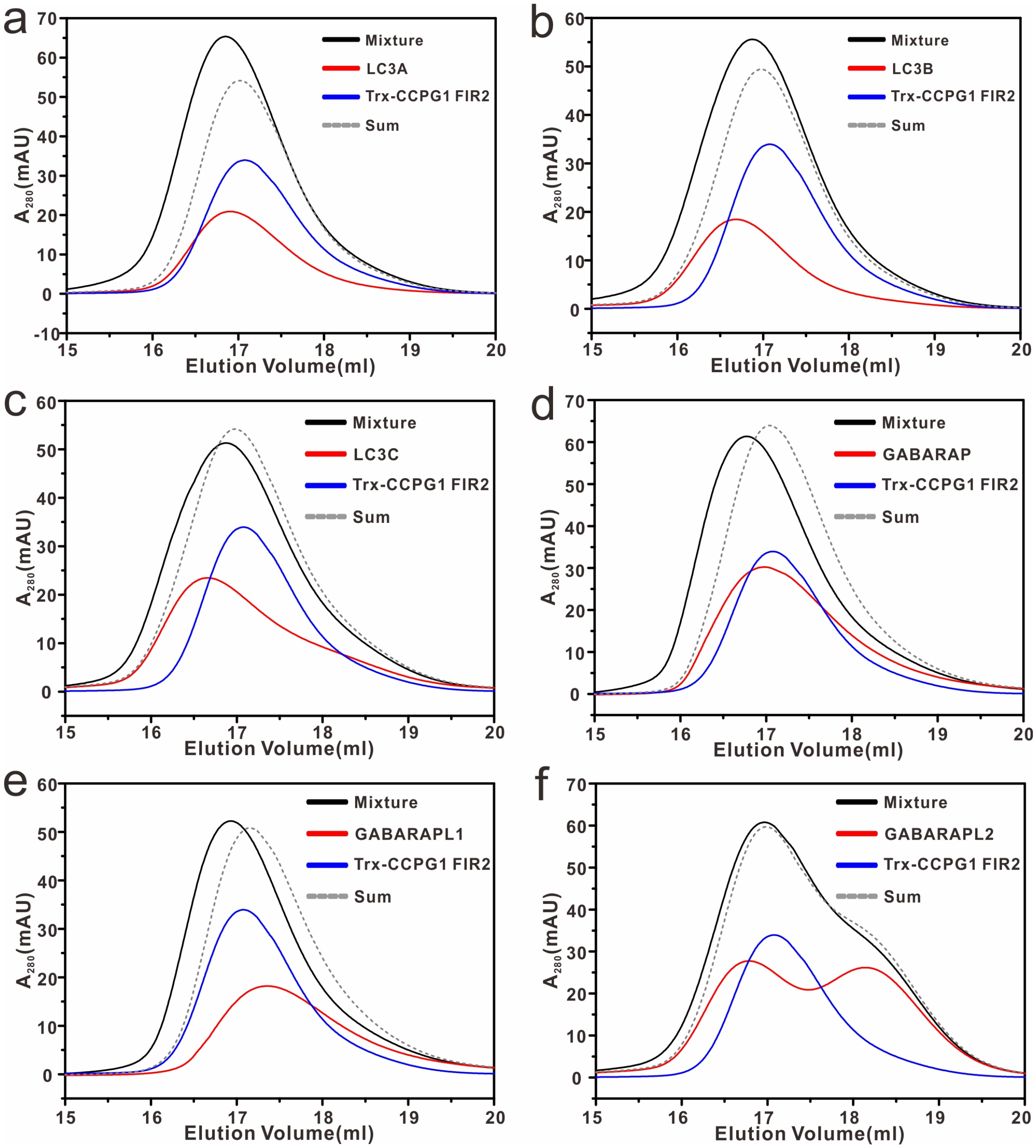

Supplementary Figure 20. Analytical gel filtration chromatography analyses of the interactions between CCPG1 FIR2 and six mammalian ATG8 family proteins. **(a-f)** Analytical gel filtration chromatography-based analyses of the interactions of CCPG1

FIR2 with LC3A (**a**), LC3B (**b**), LC3C (**c**), GABARAP (**d**), GABARAPL1 (**e**), and GABARAPL2 (**f**). These results indicate that CCPG1 FIR2 can directly bind to six different mammalian ATG8 family proteins. Source data are provided as a Source Data file.

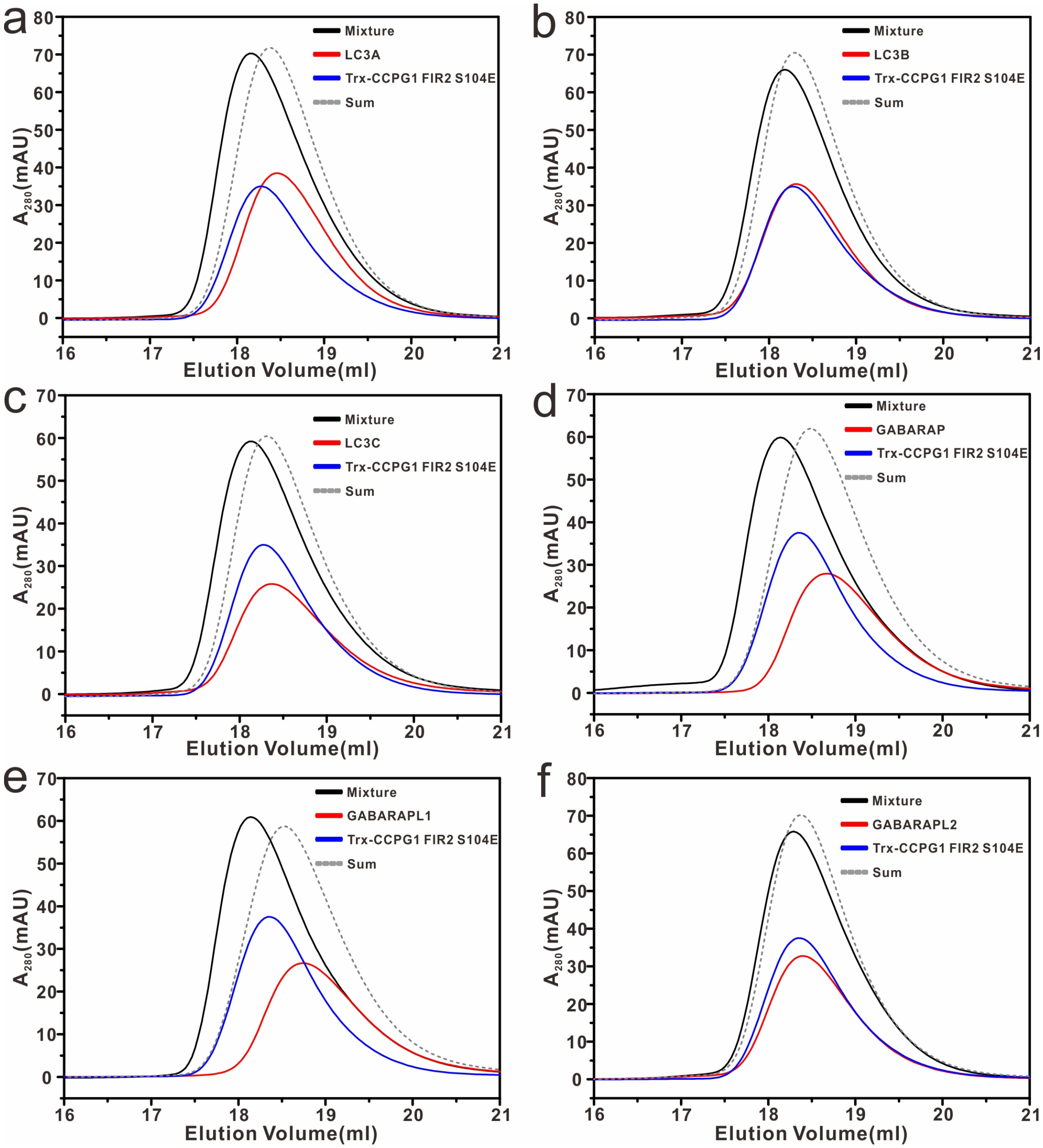

Supplementary Figure 21. Analytical gel filtration chromatography analyses of the interactions between the phosphomimetic CCPG1 FIR2 S104E mutant and six mammalian ATG8 family proteins. (**a-f**) Analytical gel filtration chromatography-based analyses of the interactions of CCPG1 FIR2 S104E mutant with LC3A (**a**), LC3B (**b**), LC3C (**c**), GABARAP (**d**), GABARAPL1 (**e**), and GABARAPL2 (**f**). Source data are provided as a Source Data file.

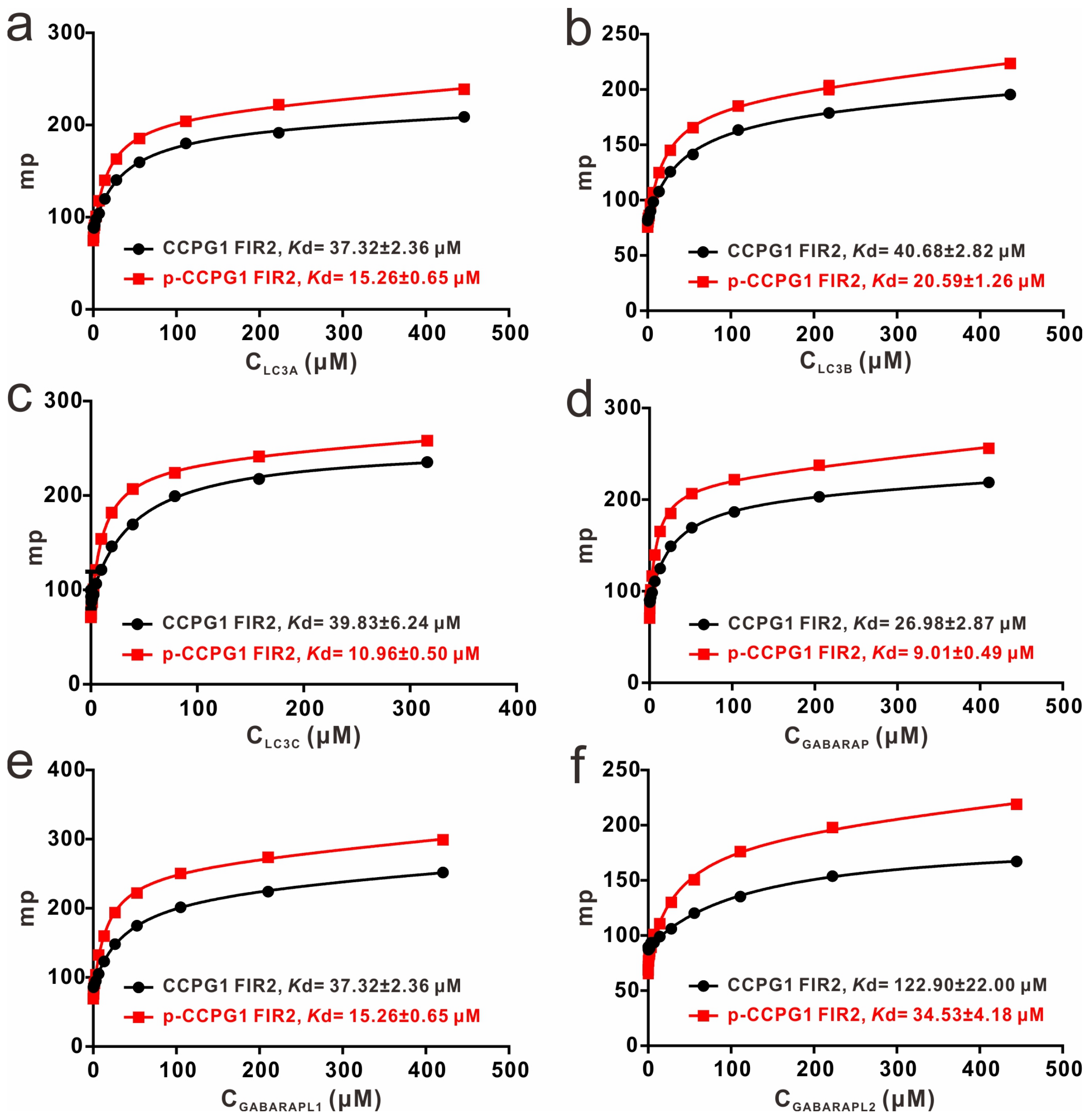

Supplementary Figure 22. FP-based measurements of the binding affinities of CCPG1 FIR2 or p-CCPG1 FIR2 with six mammalian ATG8 family proteins. (a-f) FP assay reveals the binding affinities of CCPG1 FIR2 (black line) or p-CCPG1 FIR2 (red line) with the LC3A (**a**), LC3B (**b**), LC3C (**c**), GABARAP (**d**), GABARAPL1 (**e**), and GABARAPL2 (**f**). *K*d values are the fitted dissociation constants with standard errors, when using the one-site binding model to fit the FP data. Source data are provided as a Source Data file.

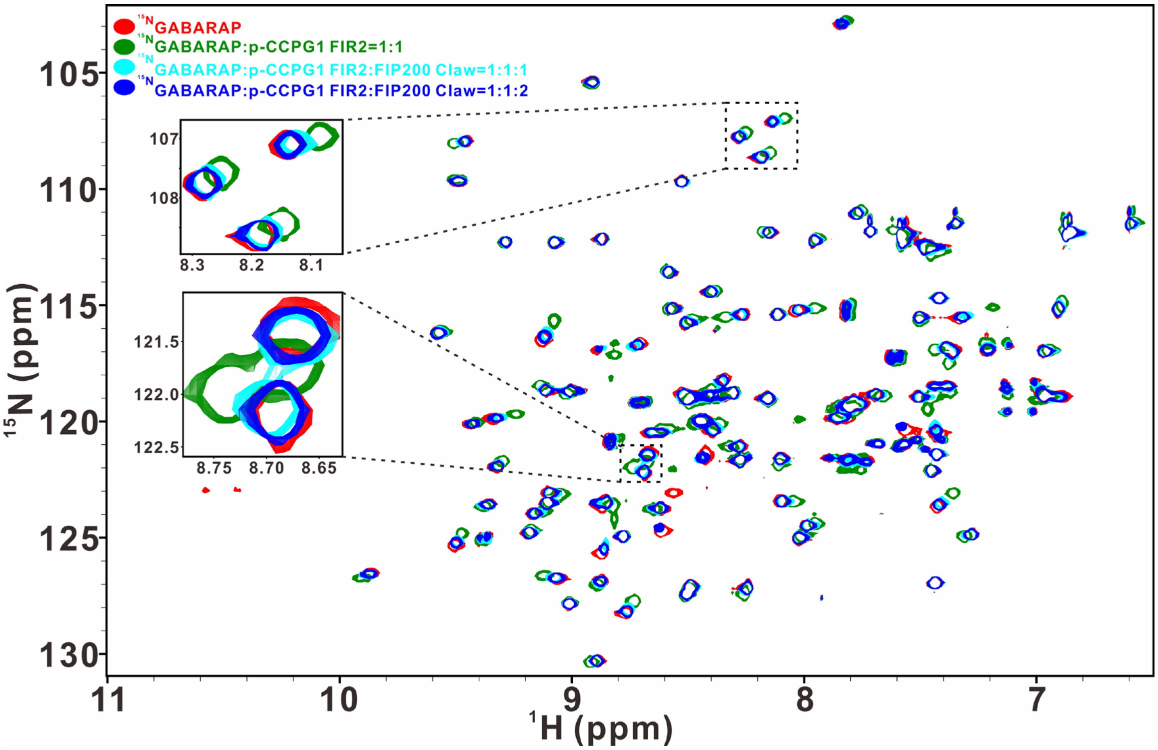

Supplementary Figure 23. NMR-based characterizations of the competitive interaction between GABARAP and FIP200 Claw for p-CCPG1 FIR2. Superposition plots of the ^{1}H-^{15}N HSQC spectra of GABARAP (red) titrated with un-labelled p-CCPG1 FIR2 peptide at the molar ratio of 1:1 (green), and then added FIP200 Claw to the mixture at the molar ratio of 1:1 (cyan), or 2:1 (blue). For clarity, the insert shows the enlarged view of a selected region of the overlaid ^{1}H-^{15}N HSQC spectra. This NMR titration result clearly demonstrated that FIP200 Claw and GABARAP are competitive in binding to p-CCPG1 FIR2.

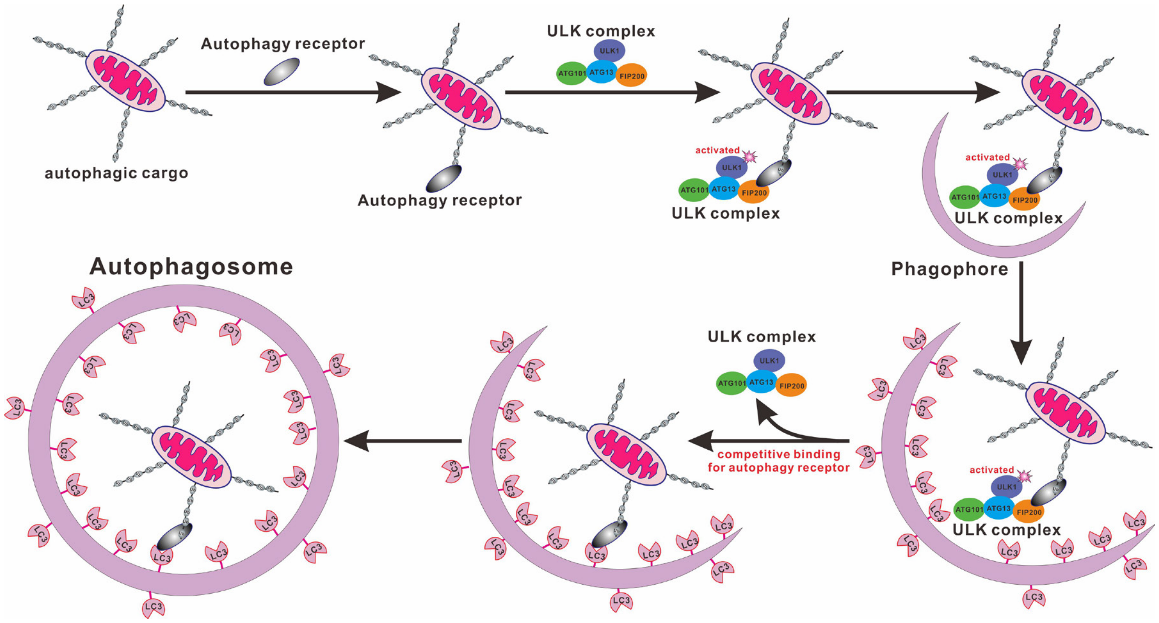

Supplementary Figure 24. A schematic cartoon diagram describing the relationship between FIR/LIR-containing autophagy receptor, ULK complex and ATG8 family proteins during the initiation of autophagosome formation in selective autophagy. In this model, LC3 stands for ATG8 family proteins.

Supplementary Table 1
Statistics of X-ray crystallographic data collection and model refinements

	FIP200(1490-1594)	FIP200(1490-1594)/p-CCPG1 FIR2 complex
Data collection		
Wavelength (Å)	0.97918	0.97876
Space group	$P2_12_12_1$	$P4_32_12$
Unit cell parameters		
a, b, c (Å)	64.544, 75.616, 99.364	51.438, 51.438, 86.151
α, β, γ (°)	90, 90, 90	90, 90, 90
Resolution range (Å)	60.17 - 1.80 (1.90 - 1.80)	50.00 - 1.40 (1.42 - 1.40)
Number of total reflections	534847	590162
Number of unique reflections	41526	23548
R_{merge} (%)[a]	8.6 (112.6)	3.7 (85.9)
$I/\sigma I$	16.7 (2.2)	98.9 (4.6)
Completeness (%)	90.6 (79.4)	99.8 (100.0)
Redundancy	12.9 (13.1)	25.1 (24.7)
Structure refinement		
Resolution (Å)	39.37 - 1.80 (1.85 - 1.80)	25.07 - 1.40 (1.46 - 1.40)
R_{work} / R_{free} (%)[b]	18.92 (30.66) / 22.95 (35.42)	18.69 (23.25) / 20.68 (25.70)
Number of reflections		
working set	41345	23439
test set	1995	1151
B-factor (Å^2)		
average	41.21	24.49
protein	40.83	22.76
RMSD bonds (Å)	0.01	0.02
RMSD angles (°)	0.80	1.49
Number of non-hydrogen atoms		
protein	3188	964
ligand	21	28
water	209	95
Ramachandran plot (%)		
favored region	99.47	98.99
allowed region	0.53	1.01
outliers	0.00	0.00

[a] $R_{merge} = \sum|I_i - I_m|/\sum I_i$, where I_i is the intensity of the measured reflection and I_m is the mean intensity of all symmetry related reflections.

[b] $R_{work} = \Sigma||F_{obs}| - |F_{calc}||/\Sigma|F_{obs}|$, where F_{obs} and F_{calc} are observed and calculated structure factors.

$R_{free} = \Sigma_T||F_{obs}| - |F_{calc}||/\Sigma_T|F_{obs}|$, where T is a test data set of about 5% of the total reflections randomly chosen and set aside prior to refinement.

Numbers in parentheses represent the value for the highest resolution shell.

Supplementary Table 2
Statistics of X-ray crystallographic data collection and model refinements

	FIP200(1490-1594)/p-Optineurin FIR complex
Data collection	
Wavelength (Å)	0.97918
Space group	*C*121
Unit cell parameters	
a, b, c (Å)	74.006, 70.631, 56.340
α, β, γ (^{o})	90.00, 109.27, 90.00
Resolution range (Å)	35.32 - 2.00 (2.07 - 2.00)
Number of total reflections	122728
Number of unique reflections	18510
R_{merge} (%)[a]	3.4 (25.4)
$I/\sigma I$	31.6 (5.8)
Completeness (%)	99.6 (99.2)
Redundancy	6.6 (6.0)
Structure refinement	
Resolution (Å)	34.96 - 2.00 (2.13 - 2.00)
R_{work} / R_{free} (%)[b]	20.28 (23.22) / 22.43 (30.51)
Number of reflections	
working set	18543
test set	884
B-factor (Å^2)	
average	56.42
protein	56.48
RMSD bonds (Å)	0.01
RMSD angles (°)	0.86
Number of non-hydrogen atoms	
protein	1739
ligand	16
water	85
Ramachandran plot (%)	
favored region	98.94
allowed region	1.06
outliers	0.00

[a] $R_{merge} = \sum|I_i - I_m|/\sum I_i$, where I_i is the intensity of the measured reflection and I_m is the mean intensity of all symmetry related reflections.

[b] $R_{work} = \Sigma||F_{obs}| - |F_{calc}||/\Sigma|F_{obs}|$, where F_{obs} and F_{calc} are observed and calculated structure factors.

$R_{free} = \Sigma_T||F_{obs}| - |F_{calc}||/\Sigma_T|F_{obs}|$, where T is a test data set of about 5% of the total reflections randomly chosen and set aside prior to refinement.

Numbers in parentheses represent the value for the highest resolution shell.

Supplementary Table 3: Current known mammalian autophagy receptors with potential FIR motifs and the relevant FIR region sequences

Protein	UniProt ID	Organism	FIR sequence	LIR sequence region
Mode I_FIRs				
P62/SQSTM1	Q13501	*H. sapiens*	CSGGDDD**W**TH**L**SSKEVDPST	331-350
STBD1	O95210	*H. sapiens*	DRVDHEE**W**EM**V**PRHSSWGDV	196-215
TRIM5	Q9C035	*H. sapiens*	KTNVLAD**F**EQ**L**RDILDWEES	178-197
FAM134B/RETREG1	Q9H6L5	*H. sapiens*	DTEEGDD**F**EL**L**DQSELDQIE	448-467
SEC62	Q99442	*H. sapiens*	SSGNGND**F**EM**I**TKEELEQQT	356-375
ATL3_LIR2	Q6DD88	*H. sapiens*	LEEKHCE**F**KQ**L**ALDHFKKTK	383-402
NLRX1	Q86UT6	*H. sapiens*	GIRTEEE**F**QL**L**HIFRRDALR	456-475
FKBP8	Q14318	*H. sapiens*	GVPPLED**F**EV**L**DGVEDAEGE	17-36
NBR1	Q14596	*H. sapiens*	SSASSED**Y**II**I**LPECFDTSR	725-744
ATL3_LIR1	Q6DD88	*H. sapiens*	QLQLFTE**Y**GR**L**AMDEIFQKP	185-204
RTN3_LIR2	O95197	*H. sapiens*	AQKPPTE**Y**SK**V**EGIYTYSLS	210-229
NDP52/CALCOCO2	Q13137	*H. sapiens*	RPENEED**I**LV**V**TTQGEVEEI	126-145
CCPG1_FIR1	Q9ULG6	*H. sapiens*	ISHEGSD**I**EM**L**NSVTPTDSC	17-36
CCPG1_FIR2	Q9ULG6	*H. sapiens*	TASDDSD**I**VT**L**EPPKLEEIG	99-118
Mode II_FIRs				
Nix/Bnip3L	O60238	*H. sapiens*	PAGLNSS**W**VE**L**PMNSSNGND	29-48
Bnip3	Q12983	*H. sapiens*	EESLQGS**W**VE**L**HFSNNGNGG	11-30
BCL2L13	Q9BXK5	*H. sapiens*	VSLGPES**W**QQ**I**AMDPEEVKS	269-288
Optineurin	Q96CV9	*H. sapiens*	SGSSEDS**F**VE**I**RMAEGEAEG	171-190
NCOA4	Q13772	*H. sapiens*	PSRIADS**F**QV**I**KNSPLSEWL	479-498
TEX264	Q9Y6I9	*H. sapiens*	SGASGSS**F**EE**L**DLEGEGPLG	266-285
FUNDC1	Q8IVP5	*H. sapiens*	YESDDDS**Y**EV**L**DLTEYARRH	11-30

The potential FIR regions of autophagy receptors in this table are categorized into two groups, Mode I and Mode II, based on the classification standards as indicated in the main text. The conserved aromatic and hydrophobic residues in the LIR core sequences are shown in bold, and the potential Ser/Thr phosphorylation sites in blue. Abbreviations: *H. sapiens, Homo sapiens.*
