# Supplementary material for: Phosphorylation regulates the binding of autophagy receptors to FIP200 Claw domain for selective autophagy initiation
Source: Nat Commun. 2021 Mar 10;12:1570. doi: 10.1038/s41467-021-21874-1 (PMC7946963; doi:10.1038/s41467-021-21874-1)
Supplement: Supplementary file 3 — Reporting Summary [file 41467_2021_21874_MOESM3_ESM.pdf]

nature research

Corresponding author(s): Lifeng Pan

Last updated by author(s): Feb 8, 2021

Reporting Summary

Nature Research wishes to improve the reproducibility of the work that we publish. This form provides structure for consistency and transparency in reporting. For further information on Nature Research policies, see our Editorial Policies and the Editorial Policy Checklist.

Statistics

For all statistical analyses, confirm that the following items are present in the figure legend, table legend, main text, or Methods section.

n/a	Confirmed	
☐	☒	The exact sample size (n) for each experimental group/condition, given as a discrete number and unit of measurement
☐	☒	A statement on whether measurements were taken from distinct samples or whether the same sample was measured repeatedly
☒	☐	The statistical test(s) used AND whether they are one- or two-sided<br>*Only common tests should be described solely by name; describe more complex techniques in the Methods section.*
☒	☐	A description of all covariates tested
☒	☐	A description of any assumptions or corrections, such as tests of normality and adjustment for multiple comparisons
☐	☒	A full description of the statistical parameters including central tendency (e.g. means) or other basic estimates (e.g. regression coefficient) AND variation (e.g. standard deviation) or associated estimates of uncertainty (e.g. confidence intervals)
☒	☐	For null hypothesis testing, the test statistic (e.g. F, t, r) with confidence intervals, effect sizes, degrees of freedom and P value noted<br>*Give P values as exact values whenever suitable.*
☒	☐	For Bayesian analysis, information on the choice of priors and Markov chain Monte Carlo settings
☒	☐	For hierarchical and complex designs, identification of the appropriate level for tests and full reporting of outcomes
☒	☐	Estimates of effect sizes (e.g. Cohen's d, Pearson's r), indicating how they were calculated

Our web collection on statistics for biologists contains articles on many of the points above.

Software and code

Policy information about availability of computer code

Data collection	We used the HKL200 software to process and scale the X-ray diffraction data. We used the UNICORN™ 6 to collect analytic gel filtration chromatography data. We used the Agilent VnmrJ 3.1 to collect NMR data. We used the SoftMax Pro 7.0 to collect fluorescence polarization assay data.
Data analysis	We used the PHENIX 1.14, Phaser 2.8.2, Coot 0.8.9.2 , MolProbity, and Pymol 1.8.7.0 to determine and analysis the three crystal structures reported in our manuscript. We used the SEDNTERP and SEDFIT16 to analysis the related AUC data in our manuscript. We used the NMRDraw 8.1 and Sparky 3.115 to analysis the NMR data in our manuscript. We used Origin8.5 to analysis the FPLC data and GraphPad Prism 6 to analysis FP data in our manuscript.

For manuscripts utilizing custom algorithms or software that are central to the research but not yet described in published literature, software must be made available to editors and reviewers. We strongly encourage code deposition in a community repository (e.g. GitHub). See the Nature Research guidelines for submitting code & software for further information.

Data

Policy information about availability of data

All manuscripts must include a data availability statement. This statement should provide the following information, where applicable:

- Accession codes, unique identifiers, or web links for publicly available datasets
- A list of figures that have associated raw data
- A description of any restrictions on data availability

The coordinates and structure factors of the FIP200(1490-1594), FIP200(1490-1594)/p-CCPG1 FIR2 complex, and FIP200(1490-1594)/p-Optineurin LIR complex determined in this study have been deposited in the Protein Data Bank under the accession code 7CZG, 7D0E and 7CZM, respectively. The source data related to

Figs. 1b, 1d, 3a-c, 3e and Supplementary Figs. 10a-f, 11a-d, 12a-d, 15a-e, 16a-f, 18a-c, 20a-f, 21a-f, 22a-f are provided as Source Data file. All additional experimental data are available from the corresponding author on request.

Field-specific reporting

Please select the one below that is the best fit for your research. If you are not sure, read the appropriate sections before making your selection.

☒ Life sciences ☐ Behavioural & social sciences ☐ Ecological, evolutionary & environmental sciences

For a reference copy of the document with all sections, see nature.com/documents/nr-reporting-summary-flat.pdf

Life sciences study design

All studies must disclose on these points even when the disclosure is negative.

Sample size	No statistical method was used to determine the correct sample size. Instead the sample size was chosen on the basis of previous experience with each specific assay. Most assays are highly reproducible when experiments are repeated on multiple occasions.
Data exclusions	No data was excluded from the study.
Replication	All biological experiments were carried out under clearly defined and standard conditions and were repeated at least twice whenever possible. All replication attempts were successful.
Randomization	The samples were randomly allocated.
Blinding	The in vitro experiments were not carried out blinded but most of them were done in parallel by at least two researchers. Blinding was not possible for all these in vitro experiments, because sample preparations and sample measurements were performed by the same individual.

Reporting for specific materials, systems and methods

We require information from authors about some types of materials, experimental systems and methods used in many studies. Here, indicate whether each material, system or method listed is relevant to your study. If you are not sure if a list item applies to your research, read the appropriate section before selecting a response.

Materials & experimental systems

n/a	Involved in the study	
☒	☐	Antibodies
☐	☒	Eukaryotic cell lines
☒	☐	Palaeontology and archaeology
☒	☐	Animals and other organisms
☒	☐	Human research participants
☒	☐	Clinical data
☒	☐	Dual use research of concern

Methods

n/a	Involved in the study	
☒	☐	ChIP-seq
☒	☐	Flow cytometry
☒	☐	MRI-based neuroimaging

Eukaryotic cell lines

Policy information about cell lines

Cell line source(s)	Sf9 cell line was sourced from ATCC
Authentication	Authentication was not performed as none of the cells used have been listed in the commonly misidentified lines
Mycoplasma contamination	Cell lines were regularly tested for mycoplasma using the MycAwayTM-Color One-Step Mycoplasma Detection Kit (YEASEN M17371)
Commonly misidentified lines (See ICLAC register)	No misidentified lines were used in this study
